# Magnesium in Obesity, Metabolic Syndrome, and Type 2 Diabetes

**DOI:** 10.3390/nu13020320

**Published:** 2021-01-22

**Authors:** Gabriele Piuri, Monica Zocchi, Matteo Della Porta, Valentina Ficara, Michele Manoni, Gian Vincenzo Zuccotti, Luciano Pinotti, Jeanette A. Maier, Roberta Cazzola

**Affiliations:** 1 Department of Biomedical and Clinical Sciences “L. Sacco”, Università di Milano, 20157 Milan, Italy; gabriele.piuri@unimi.it (G.P.); monica.zocchi@unimi.it (M.Z.); matteo.dellaporta@unimi.it (M.D.P.); valentina.ficara@unimi.t (V.F.); gianvincenzo.zuccotti@unimi.it (G.V.Z.); jeanette.maier@unimi.it (J.A.M.); 2 Department of Health, Animal Science and Food Safety, Università di Milano, 20133 Milan, Italy; michele.manoni@unimi.it (M.M.); luciano.pinotti@unimi.it (L.P.); 3 Department of Pediatrics, Ospedale dei Bambini, 2154 Milan, Italy

**Keywords:** magnesium, magnesium deficiency, magnesium supplementation, obesity, metabolic syndrome, type 2 diabetes, gut microbiota, vitamin D

## Abstract

Magnesium (Mg^2+^) deficiency is probably the most underestimated electrolyte imbalance in Western countries. It is frequent in obese patients, subjects with type-2 diabetes and metabolic syndrome, both in adulthood and in childhood. This narrative review aims to offer insights into the pathophysiological mechanisms linking Mg^2+^ deficiency with obesity and the risk of developing metabolic syndrome and type 2 diabetes. Literature highlights critical issues about the treatment of Mg^2+^ deficiency, such as the lack of a clear definition of Mg^2+^ nutritional status, the use of different Mg^2+^ salts and dosage and the different duration of the Mg^2+^ supplementation. Despite the lack of agreement, an appropriate dietary pattern, including the right intake of Mg^2+^, improves metabolic syndrome by reducing blood pressure, hyperglycemia, and hypertriglyceridemia. This occurs through the modulation of gene expression and proteomic profile as well as through a positive influence on the composition of the intestinal microbiota and the metabolism of vitamins B1 and D.

## 1. Introduction

Magnesium (Mg^2+^) is the second most abundant intracellular cation and the fourth most abundant cation of the human body. Almost all the body Mg^2+^ is found in the bones (about 60%) and soft tissues (about 40%), while <1% is in the blood. It is a cofactor of hundreds of enzymatic reactions, acting both on the enzymes as a structural or catalytic component and on the substrates. An example of Mg^2+^ bioactive activity is given by the reactions involving the complex Mg-ATP, which is an essential cofactor of kinases. For this reason, Mg^2+^ is a rate-limiting factor for many enzymes involved in carbohydrate and energy metabolism. Furthermore, Mg^2+^ is essential in the intermediary metabolism for the synthesis of the macromolecules [1]. Other vital Mg^2+^-dependent functions are muscle contraction and relaxation, normal neurological function, and release of neurotransmitters [2].

At the cellular level, Mg^2+^ homeostasis is fine-tuned by the coordinated activity of membrane channels and transporters. Some of them are ubiquitously expressed, such as transient receptor potential melastatin (TRPM) 7, Mg^2+^ transporter 1 (MagT1) and solute carrier family 41 member 1 (SLC41A1). Others are tissue-specific, such as TRPM6, expressed in the kidney and the colon, cyclin and CBS domain divalent metal cation transport mediator cyclin M2 (CNNM2), expressed in the kidney, and CNNM4, expressed in the colon [3]. 

Obesity, metabolic syndrome, and type 2 diabetes mellitus are three interrelated conditions that share a series of pathophysiological mechanisms attributable to “low-grade” systemic inflammation [4]. Mg^2+^ deficit is frequent in obese subjects [3] and is a highly prevalent condition in patients with diabetes or metabolic syndrome. Moreover, it increases the risk of developing type-2 diabetes [5]. Besides, Mg^2+^ depletion can promote chronic inflammation both directly [6,7,8] and indirectly by modifying the intestinal microbiota [9].

**Figure 1:**
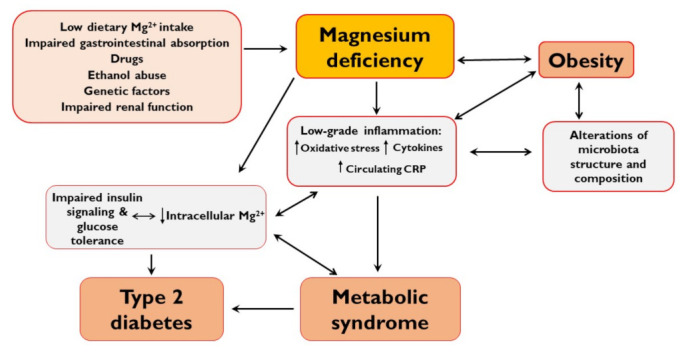
Physio-pathological mechanisms of magnesium deficiency in obesity, metabolic syndrome, and type 2 diabetes.

This review aims to offer insights into the pathophysiological mechanisms linking Mg^2+^ deficiency with obesity and the risk of developing metabolic syndrome and type 2 diabetes (Figure 1).

## 2. Mg^2+^ Deficiency

Among all the lab tests most frequently used to evaluate Mg^2+^ status in routine clinical practice is magnesemia because it is feasible and inexpensive [1,10]. However, magnesemia does not correlate with tissue pools because serum Mg^2+^ is just a tiny percentage of the intracellular/total body Mg^2+^ content [2]. This is one of the reasons why Mg^2+^ deficiency is the most underestimated electrolyte imbalance in Western countries, where a significantly high risk of latent hypomagnesemia occurs [11,12]. Based on distribution patterns of Mg^2+^ in the blood, the reference range for serum Mg^2+^ concentration is 0.75–0.95 mmol/L [13,14,15,16] and hypomagnesemia is generally defined as serum Mg^2+^ level lower than 0.7 mmol/L [3,17]. Recently, a panel of experts proposed that urinary Mg^2+^ secretion should also be considered. Specifically, a magnesemia lower than 0.82 mmol/L with Mg^2^ urinary excretion of 40–80 mg/die should be considered indicative of Mg^2^ deficiency [13]. Since serum Mg^2+^ content is only 1% of total Mg^2+^ in the body and is not representative for global intracellular Mg^2+^ status, Mg^2+^ deficiency may be underestimated and persist latently for years [16]. Subclinical hypomagnesemia is responsible for a variety of clinical manifestations that are non-specific and can overlap with symptoms of other electrolyte imbalances [18]. Some of these symptoms are depression, fatigue, muscle spasms and arrhythmias. Furthermore, a chronic low-Mg^2+^ status has been associated with an increased risk of chronic non-transmissible diseases, among which osteoporosis and sarcopenia [19,20,21]. Severe Mg^2+^ depletion, defined by serum Mg^2+^ concentration below 0.3–0.4 mmol/L, may lead to cardiac arrhythmias, tetany and seizures [3]. 

There are several causes of hypomagnesemia and one of the most relevant is an insufficient dietary intake. In fact, several studies show that the majority of the population in Europe and North America consumes less than the recommended daily allowance (RDA) of Mg^2+^, i.e., approximately 420 Mg^2+^ for adult males and 320 Mg^2+^ for adult females [22,23,24]. This deficit mainly derives from the Western-style diet (WD) that often contains only 30–50% of Mg^2+^ RDA. Indeed, the WD is based on massive consumption of processed foods, demineralized water and low amounts of vegetables and legumes, often grown in Mg^2+^-poor soil [18]. Hypomagnesemia may also be a consequence of pre-existing pathological conditions. For example, Mg^2+^ depletion is frequent in subjects affected by impaired gastrointestinal absorption caused by celiac disease [25], inflammatory bowel diseases [26,27,28] or in the presence of colon cancer, gastric bypass and other minor gastrointestinal disorders [29]. Additional causes of Mg^2+^ deficit are type 1 diabetes mellitus, renal disorders and hydro-electrolyte imbalances [30]. Hypomagnesemia is also associated, through different molecular mechanisms, with the frequent use of several medications such as diuretics (furosemide, thiazide), epidermal growth factor receptor inhibitors (cetuximab), calcineurin inhibitors (cyclosporine A), cisplatin and some antimicrobials (rapamycin, aminoglycosides antibiotics, pentamidine, foscaret, amphotericin B). It is also interesting to highlight that the wide use of proton pump inhibitors (PPI–omeprazole, pantoprazole, esomeprazole), which is generally considered safe, induces hypomagnesemia in 13% of the cases but the underlying mechanism is still unknown [31]. Moreover, ethanol abuse results in Mg^2+^ deficiency [32,33].

A low-Mg^2+^ status may also have genetic origins and derive from mutations of genes such as TRPM6, CLDN16-19 (claudin 16 and 19), KCNA1 (potassium voltage-gated channel subfamily A member 1), CNNM2 [34]. These mutations result in a severe hypomagnesemia accompanied by calcium wasting, renal failure, seizures and mental retardation [3]. Finally, from a physiological point of view, Mg^2+^ deficit may be observed after intensive sport activities with an increase in sweating, in healthy postmenopausal women [35] or during lactation [30]. Moreover, Mg^2+^ status is generally impaired in older people [36]. 

Considering the focus of this work, it is important to underline that a moderate or subclinical Mg^2+^ deficiency induces a chronic low-grade inflammation sustained by the release of inflammatory cytokines and production of free radicals, which exacerbate a pre-existing inflammatory status [7]. For this reason Mg^2+^ depletion is considered a risk factor for pathological conditions characterized by chronic inflammation, such as hypertension and cardiovascular disorders but also metabolic syndrome and diabetes [29,37,38].

## 3. Mg^2+^ and Obesity

Obesity and its comorbidities, including metabolic syndrome and type 2 diabetes, are a relevant medical problem worldwide. Obesity is the result of unhealthy diets, high in calories, but poor in essential nutrients. As a consequence, obese subjects are often Mg^2+^ deficient [39]. Indeed, the National Health and Nutrition Examination Survey (NHANES) 3 study underlines that Mg^2+^ deficit is more prevalent in subjects with body mass index (BMI) in the obese range than in the normal American population [40,41]. Analogously, Mg^2+^ intake is impaired in 35% of French individuals with BMI > 35 kg/m^2^ [42]. The 30-year longitudinal CARDIA study, performed on more than 5000 subjects, indicates that Mg^2+^ intake is inversely associated with the incidence of obesity and with the levels of C reactive protein [43]. Besides, in animal models of diet-induced obesity Mg^2+^ supplementation prevents the accumulation of adipose tissue [44] and human studies report an inverse association between Mg^2+^ intake and markers of adiposity, such as BMI and waist circumference [45,46,47].

In obese subjects, most of the energy of the diet derives from refined grains and simple sugars and, consequently, their hepatic glucose catabolism is very active. Several key enzymes of glucose oxidation pathways are Mg^2+-^dependent and Mg^2+^ is necessary also for the activation of vitamin B1 into thiamine diphosphate (TDP) that is another critical coenzyme of oxidative metabolism. Importantly, TDP-dependent enzymes require Mg^2+^ to reach optimal activation [48]. Therefore, low intracellular concentrations of Mg^2+^ and/or TDP may alter the oxidative metabolism of glucose. In the liver, a decrease of the activity of the Mg^2+^- and TDP-dependent enzyme pyruvate dehydrogenase may divert glucose metabolism into the oxidative phase of the pentose phosphate pathway, thus generating an excess of NADPH [48]. NADPH provides essential redox potential for synthetic pathways, including fatty acid biosynthesis, thus promoting an increased synthesis of triglycerides and very low-density lipoprotein and, consequently, a higher triglyceride storage in adipocytes that increases the extent of obesity and the risk of obesity co-morbidities such as dyslipidemia, metabolic syndrome and type 2 diabetes [49,50,51]. 

Moreover, obese subjects are often deficient also in vitamin D [49,52] both in the presence and in the absence of type 2 diabetes [53], and Mg^2+^ is essential also for vitamin D synthesis and activation [54]. A randomized controlled trial suggests that optimal Mg^2+^ status may be fundamental for optimizing vitamin D status [55]. Because of its role in the renin-angiotensin system and its immunomodulatory properties, vitamin D deficiency is identified as a potential risk factor in cardiometabolic disorders, including insulin resistance, metabolic syndrome and cardiovascular diseases [56]. Moreover, chronic latent Mg^2+^ deficiency and/or Vitamin D deficiency predispose non-diabetic obese subjects to an increased risk of cardiometabolic diseases. Meanwhile, maintaining a normal Mg^2+^ status improves the beneficial effect of Vitamin D on cardiometabolic risk indicators [57]. Interestingly, an interventional study performed on healthy women showed a significant increase in serum concentration of Mg^2+^ in obese but not in non-obese subjects after vitamin D intramuscular injection, probably caused by increased Mg^2+^ renal retention induced by vitamin D and emphasized by baseline Mg^2+^ deficiency of the obese subjects [58].

## 4. Mg^2+^ in Metabolic Syndrome

Obesity and metabolic syndrome (MetS) are both characterized by excessive accumulation of body fat. However, while obesity only implies the accumulation of excess body fat, metabolic syndrome is a disorder of accumulation and use of energy, promoted by low-grade systemic inflammation, and resulting in central adiposity, hypertension, dyslipidemia, or insulin resistance. Many studies have found a positive correlation between low dietary Mg^2+^ intake and MetS risk independently from other risk factors such as age, gender, BMI, race, educational attainment, marital status, smoking, alcohol intake, exercise, energy intake, percentage of calories from saturated fat, use of an antihypertensive or lipid medication [59,60,61,62,63,64]. Dibaba et al. showed in the last meta-analysis available that the dietary Mg^2+^ intake is inversely associated with the prevalence of MetS [63]. A recent cross-sectional analysis performed in a large Chinese population reports an inverse correlation between dietary Mg^2+^ intake and the prevalence of MetS [65]. In more than 11.000 middle-aged and older women high dietary Mg^2+^ intake lowers systemic inflammation and the risk of the MetS [59]. An interesting Serbian study shows a positive association between chronic exposure to insufficient Mg^2+^ in drinking municipalities water and the prevalence of hypertension and MetS [66].

MetS exponentially increases the risk of developing type 2 diabetes, cardiovascular disease and, in general, morbidity and mortality. Proper Mg^2+^ intake reduces cardiometabolic risk and is associated with a reduced hazard of cardiovascular disease, diabetes, and all-cause mortality [67,68,69,70]. Likewise, higher levels of circulating Mg^2+^ are associated with a lower risk of cardiovascular disease, mainly coronary artery disease [71].

Low chronic Mg^2+^ dietary intake leads to serum and intracellular Mg^2+^ deficiency. This is particularly evident in obese people with MetS, in elderly subjects and non-white people with insulin resistance [72,73,74].

Mg^2+^ is a natural calcium (Ca^2+^) antagonist, and its metabolic effect needs to be discussed according to Ca^2+^ concentration. A recent meta-analysis suggests that high Ca^2+^ dietary intake reduces the risk of MetS [75]. Other experimental data suggest that a higher Ca^2+^/Mg^2+^ intracellular ratio, induced by a diet high in Ca^2+^ and low in Mg^2+^, may lead to hypertension, insulin resistance, and MetS [76]. Accordingly, subjects who meet the recommended daily allowance for both Mg^2+^ and Ca^2+^ have reduced risk of MetS [76]. Mg^2+^ and Ca^2+^ work together to regulate the metabolic response of overweight and obese subjects, and an unbalanced Ca^2+^/Mg^2+^ ratio maximizes the effect of their single deficiency. The optimal Ca^2+^/Mg^2+^ ratio leads to the best-decreased risk of MetS [77,78]. 

## 5. Mg^2+^ in Type 2 Diabetes

**Figure 2:**
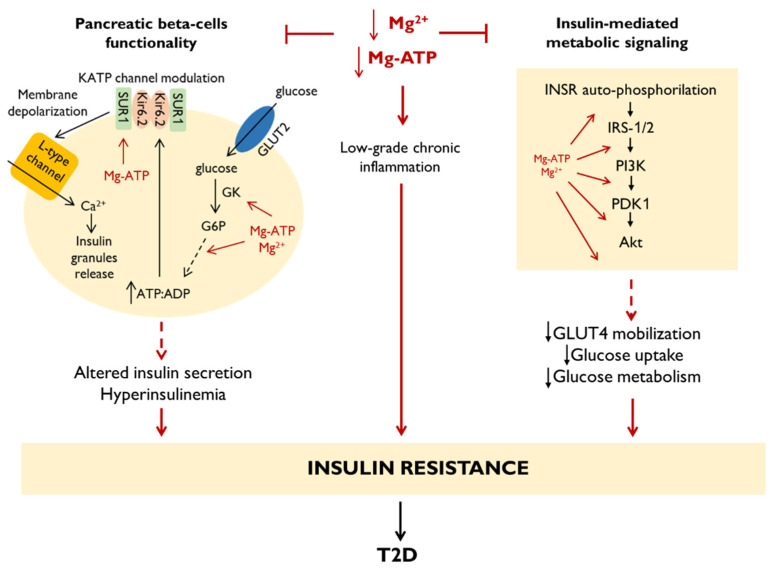
Links between Mg^2+^ and insulin signaling. For details, please see the text.

Type 2 diabetes (T2D) is often associated with altered Mg^2+^ homeostasis and Mg^2+^ intake is inversely associated with the risk of T2D in a dose-response manner [79,80]. Epidemiologic studies have shown a high prevalence of hypomagnesemia in subjects with T2D [81,82]. Mg^2+^ depletion in patients with T2D is mainly caused by a low intake and an increased urinary loss of Mg^2+^, probably resulting from impaired renal function [82]. Moreover, recent findings demonstrate that hypomagnesemia is strongly associated with the progression of T2D [83]. In particular, if it is true that insulin regulates Mg^2+^ homeostasis, at the same time Mg^2+^ is also a significant determinant of post-receptor insulin signaling. The influence of Mg^2+^ on glucose metabolism, insulin sensitivity, and insulin action could explain the negative association between Mg^2+^ intake and T2D incidence [82,84,85,86,87] (Figure 2). To better understand this issue, it is worth recalling that insulin secretion is started by a Ca2+ influx that is competitively inhibited by extracellular Mg^2+^ and, consequently, insulinemia is inversely correlated with magnesemia. Circulating glucose is easily taken from cells β through the glucose transporter 2 (GLUT2), and then converted in glucose-6-phosphate (G6P) by glucokinase (GK). The oxidation of G6P in glycolysis determines an increase in the ATP/ADP ratio leading to the closure of ATP-sensitive K^+^ channels (KATP channels) and, consequently, to the depolarization of the membrane, followed by the opening of voltage-dependent Ca^2+^ channels [88]. The increase in intracellular concentrations of Ca^2+^ triggers the fusion of insulin-containing granules with the membrane and the subsequent release of their content. The molecular mechanisms by which Mg^2+^ contributes to insulin resistance are mostly unrevealed. However, it is accepted that Mg^2+^ deficiency has a significant impact on insulin secretion and may contribute to dysfunction of pancreatic beta cells in T2D [89]. This depends on the key roles played by Mg^2+^ in the glucose-dependent signaling inducing insulin release. The activities of GK and many glycolytic enzymes depend on Mg-ATP complex, thus, a low intracellular Mg^2+^ concentration results in decreased ATP level in the cells. In addition, the closure of KATP channels depends on ATP binding to the Kir6.2 subunit, while the opening of these channels depends on Mg-ATP binding to the SUR1 subunit. The reduction in the intracellular levels of both ATP and Mg-ATP deranges the fine regulation of KATP channels. This leads to an increase in the basal secretion of insulin and induces hyperinsulinemia, thus contributing to a chronic exposure of cells to insulin and to the development of insulin resistance fostered also by the concomitant low grade inflammation [89]. Moreover, the prolonged hyperinsulinemia typical of insulin resistance induces an increase in renal excretion of Mg^2+^, thus perpetuating a vicious cycle [90]. In addition, we recall that physiological concentrations of insulin and glucose stimulate Mg^2+^ transport, thus increasing intracellular Mg^2+^ content. It is noteworthy that low intracellular Mg^2+^ impairs cell responsiveness to insulin, because low intracellular Mg^2+^ alters the tyrosine-kinase activity of the insulin receptor (INSR), leading to the development of post-receptor insulin resistance and decreased cellular glucose utilization [89,91]. In particular, Mg^2+^ and Mg-ATP complex are key regulators of the PI3K/Akt kinase pathway downstream to the INSR. This pathway starts with INSR auto-phosphorylation, which triggers the downstream kinase cascade. Insulin receptor substrate (IRS) mainly activates phosphatidylinositol-4,5-bisphosphate-3-kinase (PI3K), which generates the second messenger phosphatidylinositol-3,4,5-triphosphate (PIP3). PIP3 activates 3-phosphoinositide dependent protein kinase-1 (PDK1), which activates Akt. Akt regulates the metabolic actions of insulin, including glucose uptake by GLUT4 mobilization in skeletal muscle and adipose tissue, glycogen and protein synthesis and lipogenesis. For this reason, the lower is the basal intracellular Mg^2+^ concentration, the higher is the amount of insulin required to metabolize the same glucose load, indicating decreased insulin sensitivity [89,91]. All these data underline that insulin action is strictly dependent on the intracellular Mg^2+^ concentration. 

Mg^2+^ deficiency can also contribute to T2D through the modulation of Na^+^/K^+^-ATPase that is crucial for maintaining the membrane potential and low cytoplasmic sodium concentration. Mg^2+^ ions drive the conformational change of the sodium pump whose dysfunction has been correlated to T2D [92,93]. Moreover, some single nucleotide polymorphisms in the TRPM6 gene are associated with an increased risk of developing T2D because TRPM6 cannot be activated by insulin in the presence of these mutations [94]. 

The observation that several pharmacological treatments for diabetes, such as metformin, appear to increase Mg^2+^ levels further supports this assumption and suggests a substantial interdependence between Mg^2+^ deficiency and the development of insulin resistance and T2D. Mg^2+^ deficiency may not be a secondary consequence of T2D, but it may contribute to insulin resistance and altered glucose tolerance, thereby leading to T2D [82].

Few studies have discussed the relationship between hypomagnesemia and metabolic disorders in childhood and adolescence. Mg^2+^ deficiency in obese children may be secondary to decreased dietary Mg^2+^ intake. Obese children show lower serum Mg^2+^ levels than the normal-weight control group. In obese children and adolescents, Mg^2+^ blood concentration is inversely correlated with the degree of obesity and is related to an unfavorable serum lipid profile and higher systemic blood pressure than healthy controls [95,96]. The association between Mg^2+^ deficiency and insulin resistance has been described also in childhood [97]. Mg^2+^ supplementation or increased intake of Mg^2+^-rich foods to correct its deficiency may represent an essential and inexpensive tool in preventing T2D in obese children.

## 6. Mg^2+^ and Gut Microbiota

The gut microbiota is a complex microbial ecosystem, symbiotic with humans, that plays a crucial role in a series of pathophysiological processes. In healthy subjects the gut microbiota is rich in microbial species and, through its genes and metabolites (i.e., short-chain fatty acids, amino acid derivatives, secondary bile acid), it acts as an immunologic and metabolic organ [98,99]. By contrast, obesity and related metabolic disorders, such as MetS and T2D, determine profound functional and compositional alterations in the intestinal microbiota, collectively referred to as dysbiosis [100].

Little is known about Mg^2+^ deficiency and gut microbiota in humans, while some data are available in animal models. A 6-week Mg^2+^-deficient diet in rodents altered the gut microbiota and was associated with anxiety-like behavior [101]. In particular, Mg^2+^ deficiency may mediate an imbalance of the microbiota–gut–brain axis, which contributes to the development of depressive-like behavior [102]. It should be pointed out that obesity increases the risk of depression and depression was found to be predictive of developing obesity [103]. Moreover, epidemiological data have demonstrated that obesity is an important risk factor for the development of gastroesophageal reflux disease [104] and PPI used for the treatment of such disease, may lead to Mg^2+^ deficiency also through the involvement of the gut microbiome [105]. As previously mentioned, Mg^2+^ deficiency is a nutritional disorder connected to a low-grade, latent chronic inflammatory state. Interestingly, in Mg^2+^-deficient mice, changes in intestinal bifidobacteria levels are associated with an inflammatory response, thus creating an effective link between Mg^2+^ status, gut microbiota and inflammation [106]. 

It is now widely accepted that an altered gut microbiota composition participates in systemic low-grade inflammation [107,108,109,110]. An analysis of patients with different glucose tolerance suggests that both structure and diversity of gut microbiota are altered in the presence of impaired glucose regulation and T2D [111,112]. However, Thingholm et al. compared the microbiota composition of obese versus lean subjects and obese versus obese with T2D. The authors observed that microbiome diversity and functionality were significantly reduced in obese compared to lean subjects, while only modest differences emerged when comparing the microbiome of obese versus obese with T2D [113]. Therefore, the development of obesity-associated T2D could be related to a progressive disruption of the gut microbiome. Gut microbiota manipulation through dietary adjustment has become an important research direction in T2D prevention and therapy. In this perspective, Mg^2+^ supplementation might help in remodeling the microbiota. Indeed, Mg^2+^ supplementation in obese subjects with and without T2D affects microbial composition and functional potential [113]. Moreover, dietary supplementation with a multi-mineral functional food derived from seaweed and seawater, rich in bioactive Mg^2+^ and other trace elements, significantly enhances the gut microbial diversity in adult male rats [114].

To conclude, an adequate Mg^2+^ dietary intake could positively affect the composition of the intestinal microbiota and, consequently, the host metabolism, thus helping in preventing metabolic alterations associated with the development of MetS and TD2. However, the path for clarifying the impact of Mg^2+^ in this emerging field of research is still long. 

## 7. Dietary Mg^2+^

The intakes of food rich in Mg^2+^, including whole grains, nuts and seeds, legumes, and dark-green vegetables, were associated with a lower incidence of obesity, T2D and MetS [43]. Therefore, correcting unhealthy diets is a priority to meet the daily-recommended requirement for Mg^2+^. However, because of agronomic and environmental factors as well as food processing, Mg^2+^ content in fruits and vegetables dropped in the last 50 years [115] and it might be necessary to supplement it. This is an approach that has been proven beneficial in T2D and MetS (Figure 2). The daily administration of 250 mg of elemental Mg^2+^ for three months improves glycemic control in T2D subjects as demonstrated by the significant reduction of glycated hemoglobin, insulin levels, C-peptide, and Homeostatic Model Assessment for Insulin Resistance (HOMA-IR) [116]. This effect is probably due to the correction of an underlying latent Mg^2+^ deficiency. Indeed, the supplementation with 360 mg of Mg^2+^ for the same period does not improve insulin sensitivity in normomagnesemic T2D patients [117]. The administration of 250 mg of elemental Mg^2+^ for 12 weeks improved the wound healing of diabetic foot ulcers, decreasing the lesion size, and ameliorating glucose metabolism [118]. Mg2+ could also affect glucose metabolism by modulating the concentration of inflammatory cytokines, such as IL-6. Although these data need to be confirmed, in prediabetic subjects, the supplementation with 380 mg of Mg2+ provides a trend of reduction in IL-6 plasmatic levels while there are no differences in the levels of C-reactive protein (CRP), Tumor Necrosis Factor-alpha (TNF-alpha), and Interleukin 10 (IL-10) [119]. It is noteworthy that, in apparently healthy runners fed a low Mg2+ diet, the administration of 500 mg of Mg2+ lowers IL-6 levels, reduces muscle soreness and increases post-exercise blood glucose [120].

Mg^2+^ supplementation seems to improve blood pressure control and vascular resistance in patients with essential hypertension [121]. The administration of 300 mg of Mg^2+^ for one month decreases systolic and diastolic pressures, systemic vascular resistance, and left cardiac work [122]. The oral Mg^2+^ supplementation with 600 mg for 12 weeks is associated with moderate but consistent ambulatory blood pressure reduction in patients with mild hypertension [123]. This result can be explained by the evidence that Mg^2+^ is a Ca^2+^ antagonist, increases the synthesis of vasodilators such as prostacyclin and nitric oxide, and inhibits vascular calcifications through the modulation of TRPM7 [123,124]. An increase in the transcription of the Mg^2+^ channel TRPM6 could explain the antihypertensive effects of Mg^2+^ supplementation. The increase of TRPM6 mRNA expression is obtained with the administration of 360 mg of Mg^2+^ for four months [124]. The positive effect of Mg^2+^ supplementation on blood pressure is also reported in patients already undergoing drug treatment for hypertension. In thiazide-treated women, the administration of 600 mg of Mg^2+^ improves endothelial function and subclinical atherosclerosis [125]. In hemodialysis patients, the administration of 440 mg of Mg^2+^ for six months decreases carotid intimate-media thickness, which is a marker of cardiovascular disease. This effect is not associated with an improvement of endothelial function measured by brachial artery flow-mediated dilatation and might be explained by the modulation of calcification through the regulation of calcium and phosphorus concentration in blood [126]. In disagreement with the aforementioned results, a randomized controlled trial on overweight and obese middle-aged and elderly adults did not report any improvement of endothelial function and cardiometabolic risk markers after supplementing 350 mg Mg^2+^ daily for 24 weeks [126,127].

Since correcting Mg^2+^ status lowers blood pressure, corrects lipid profile and ameliorates the control of glycemia, it is not surprising that Mg^2+^ supplementation has positive effects in MetS. The supplementation of 380 mg of Mg^2+^ for 16 weeks improves MetS by reducing blood pressure, hyperglycemia, and hypertriglyceridemia [128], because the correction of hypomagnesemia leads to changes in gene expression and proteomic profiling consistent with favorable effects on several metabolic pathways [85]. The effect of Mg^2+^ on the lipid profile is still debated and appears to be mediated by the improvement of insulin resistance and appears to be present only if Mg^2+^ supplementation corrects a previous deficiency [129]. The administration of 370 mg of Mg^2+^ in healthy normomagnesemic young men with a family history of MetS does not show beneficial effects on blood pressure, vascular function, and glycolipid profile [130]. For MetS, as well as for T2D, the positive effect of Mg^2+^ administration is only registered if the supplementation corrects a condition of hypomagnesemia.

**Figure 3:**
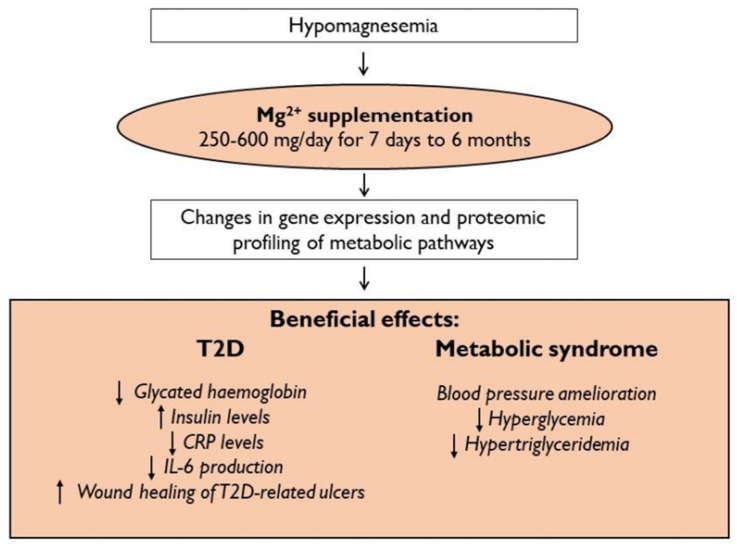
Beneficial effects of magnesium supplementation in hypomagnesemic patients with metabolic syndrome and type 2 diabetes.

Some critical issues emerge from the analysis of the literature and complicate the interpretation of the data (Table 1). First, there is no agreement on the dosages and timing of Mg^2+^ supplementation in the treatment of MetS, T2D, and hypertension. Considering the literature just discussed [109,110,111,112,113,114,115,116,117,118,119,120,121,122,123,124], the dosage of Mg^2+^ varies from 250 mg to 600 mg, with a median of 380 mg (95% confidence interval (CI) 300–500 mg). The time of Mg^2+^ supplementation ranges between 7 days and six months, with a median of about three months (95% CI 4–24 weeks). Besides, there is no consensus on the type of Mg^2+^ salt to use. The bioavailability of different Mg^2+^ salts has been investigated in depth [131,132,133,134]. Magnesium sulfate, oxide, carbonate, chloride, citrate, malate, acetate, gluconate, lactate, aspartate, fumarate, acetyl taurate, bis-glycinate, and pidolate are all employed in Mg^2+^ supplementation. In part, the differences in the bioavailability of Mg^2+^ salt is due to their different solubility [135]. Although organic Mg^2+^ salts were slightly more bioavailable than inorganic Mg^2+^ salts, inorganic Mg^2+^ salts have been administered to patients with interesting clinical outcomes. The choice of the type of Mg^2+^ salt based on its bioavailability conditions its dosage and the possible side effects, especially as intestinal symptoms of osmotic dysentery [136] (Figure 3).

## 8. Conclusions

Obesity, type 2 diabetes, and metabolic syndrome are intertwined conditions characterized by chronic low-grade inflammation partly attributable to Mg^2+^ deficiency. In metabolic diseases, a low Mg^2+^ status mainly due to unhealthy diets contributes to generate a pro-inflammatory environment that exacerbates metabolic derangement. Mg^2+^ supplementation seems to foment the correction of this vicious loop, but at the moment it is hard to interpret whether Mg^2+^ beneficial effects occur through a direct effect on metabolic pathways or an indirect action on inflammation, or both.

Several important points need to be clarified. At the clinical level, more studies are necessary to define which Mg^2+^ salt and which dosage guarantee better outcomes. In addition, the investigation of microbiota in hypomagnesemic subjects might provide interesting hints and suggest targeted dietary approaches aimed at harmonizing the gut microbial ecosystem. In addition, biomarkers that grant the possibility of evaluating Mg^2+^ homeostasis should be identified. At the cellular and molecular level, it is important to focus on the role of intracellular Mg^2+^ in modulating cell function, from the regulation of metabolism to the release of inflammatory mediators.

Considering the worldwide prevalence of obesity, type 2 diabetes and metabolic syndrome, the correction of bad dietary habits and, eventually, the supplementation of Mg^2+^ might represent an inexpensive but valuable tool to contain the occurrence and the progression of these conditions.

## Figures and Tables

**Table 1:** A quick recap of the last and most relevant clinical trials describing the effects of Mg^2+^ supplementation on obesity, metabolic syndrome (MetS), and type 2 diabetes (T2D).

Author(s)	Year	Dosage of Mg^2+^ Supplementation	Type of Salt	Timing of Mg^2+^ Supplementation	Effects of Mg^2+^ Supplementation	Ref.
Elderawi WA et al.	2018	250 mg/day	Oxide, gluconate, lactate	3 months	Improves glycemic control in T2D subjects with a reduction of glycated hemoglobin, insulin levels, C-peptide, and HOMA-IR.	[116]
Navarrete-Cortes A et al.	2014	360 mg/day	Lactate	3 months	No effects on insulin sensitivity.	[117]
Razzaghi R et al.	2018	250 mg/day	Oxide	12 weeks	Improves wound healing of diabetic foot ulcers, decreasing the lesion size, and ameliorating glucose metabolism.	[118]
Simental-Mendía LE et al.	2012	380 mg/day	Chloride	3 months	Reduces IL-6 plasmatic levels.	[119]
Steward CJ et al.	2019	500 mg/day	Oxide, stearate	7 days	Lowers IL-6 levels, reduces muscle soreness and increases post-exercise blood glucose.	[120]
Banjanin N et al.	2018	300 mg/day	Oxide	1 month	Decreases systolic and diastolic pressures, systemic vascular resistance, and left cardiac work.	[122]
Hatzistavri LS et al.	2009	600 mg/day	Pidolate	12 weeks	Reduces ambulatory blood pressure.	[123]
Rodríguez-Ramírez M et al.	2017	360 mg/day	Lactate	4 months	Increases TRPM6 mRNA relative expression.	[124]
Cunha AR et al.	2017	600 mg twice a day	Chelate (not better specified)	6 months	Improves endothelial function and subclinical atherosclerosis.	[125]
Mortazavi M et al.	2013	440 mg 3 times per week	Oxide	6 months	Decreases carotid intimate-media thickness, which is a marker of cardiovascular disease.	[126]
Joris PJ et al.	2017	350 mg/day	Citrate	24 weeks	No effect on endothelial function.	[127]
Rodríguez-Morán M et al.	2018	380 mg/day	Chloride	16 weeks	Improves MetS by reducing blood pressure, hyperglycemia, and hypertriglyceridemia.	[128]
Cosaro E et al.	2014	370 mg twice a day	Pidolate	8 weeks	Effects on blood pressure, vascular function, and glycolipid profile.	[130]

HOMA-IR: Homeostasis Model Assessment-estimated for Insulin Resistance; IL-6: Interleukin-6; TRPM6: Transient Receptor Potential Melastatin 6.

## Data Availability

Data sharing is not applicable to this article.
